# Disrupted prefrontal-sensorimotor functional connectivity mediates the association between depression and symptom severity in patients with irritable bowel syndrome

**DOI:** 10.3389/fnhum.2026.1817128

**Published:** 2026-04-14

**Authors:** Xi Liu, Mengqi Wang, Wei Niu, Peicai Li, Qingshuang Jiang

**Affiliations:** 1 Department of Gastroenterology, Tianjin Integrated Traditional Chinese and Western Medicine Hospital, Tianjin Nankai Hospital, Tianjin University, Tianjin, China; 2 Tianjin Key Laboratory of Acute Abdomen Disease Associated Organ Injury and ITCWM Repair, Tianjin Integrated Traditional Chinese and Western Medicine Hospital, Tianjin Nankai Hospital, Tianjin University, Tianjin, China; 3 Xingnan Street Community Health Service Center, Nankai District, Tianjin, China; 4 Tianjin Medical University, Tianjin, China; 5 Tianjin Medical University Chu Hsien-I Memorial Hospital (Metabolic Diseases Hospital), Tianjin, China; 6 Tianjin Medical University General Hospital Airport Hospital, Tianjin, China

**Keywords:** brain-gut-axis, depression, functional connectivity, irritable bowel syndrome, resting-state fMRI

## Abstract

**Background:**

Depressive symptoms are well-established psychological comorbidities significantly associated with the onset and progression of irritable bowel syndrome (IBS). Although alterations in resting-state functional connectivity (rsFC) have been shown in IBS, the potential mediating role of aberrant rsFC in the relationship between depressive symptoms and IBS severity remains unclear.

**Methods:**

This exploratory resting-state functional magnetic resonance imaging (rs-fMRI) study recruited participants including 32 IBS patients and 37 HCs. We employed whole-brain functional connectivity strength (FCS) analysis to identify regions with significant rsFC alterations. Subsequently, seed-based whole-brain FC analysis (using FCS-altered regions as seeds) and seed-to-seed FC analysis (based on the AAL atlas) were performed to examine group differences in FC. The mean rsFC value within clusters with significant between-group differences were correlated with clinical scores. Mediation models were constructed to test the mediating role of aberrant FC in the relationship between depressive symptoms and IBS severity.

**Results:**

Compared to HCs, IBS patients exhibited significantly reduced FCS in the bilateral prefrontal cortex (PFC). Seed-based whole-brain FC analysis revealed that, relative to HCs, IBS patients had increased FC between the left PFC and primary sensory cortex (SI), but decreased FC between the left PFC and right PFC, as well as between the left PFC and bilateral thalamus. Seed-to-seed FC analysis further showed aberrant connectivity within the default mode network (DMN) in IBS patients, with the left angular gyrus (a key DMN hub) demonstrating disrupted connectivity with multiple regions (e.g., right PFC, left precentral gyrus, and SI). Correlation analyses indicated that left PFC FCS was positively correlated with Self-Rating Depression Scale (SDS) scores and negatively correlated with Irritable Bowel Syndrome Symptom Severity Scale (IBS-SSS) scores in IBS patients; additionally, SDS scores were positively correlated with IBS-SSS scores. Mediation analysis confirmed that left PFC FCS significantly mediated the relationship between SDS (predictor) and IBS-SSS (outcome), while the reverse model showed no significant mediating effect.

**Conclusions:**

Our exploratory findings suggest that depressive symptoms in IBS are associated with alterations in functional connectivity (FC), particularly within the prefrontal cortex. Crucially, the identified FC changes might be a potential interplay between depression and symptom severity in IBS.

## Background

Irritable bowel syndrome (IBS) is a widespread functional gastrointestinal disorder, affecting an estimated over 10% of the global population (Drossman et al., 2002). It is clinically defined by chronic and recurrent abdominal pain or discomfort, accompanied by alterations in bowel habits (Ford et al., 2020). Beyond its symptomatic burden, IBS considerably diminishes quality of life and imposes a substantial healthcare burden (Black and Ford, 2020). A notable clinical feature of IBS is its strong link with depression (Fond et al., 2014). Affected individuals exhibit significantly higher depressive symptoms compared not only to healthy controls but also to those with other bowel conditions, such as inflammatory bowel disease (IBD). For instance, Midenfjord et al. (2019) demonstrated through univariate analysis that IBS patients experiencing psychological distress report more severe gastrointestinal symptoms (Midenfjord et al., 2019). Correspondingly, Lee et al. (2017) observed that severe depressive symptoms were associated with elevated odds ratios for IBS (Lee et al., 2017). Further empirical support indicates that depressive psychopathology doubles the likelihood of developing gastrointestinal symptoms in IBS (Choi et al., 2017; Jang et al., 2017). Unraveling the pathophysiology of IBS is essential for identifying reliable diagnostic biomarkers and advancing new treatment strategies. Nonetheless, the precise mechanisms underlying the disorder remain elusive.

In the absence of identifiable organic pathology, IBS is classified as a functional disorder, believed to originate from dysregulated brain–gut communication (Sarnoff et al., 2023; Yuan et al., 2023). Functional neuroimaging offers a means to quantify central processing of viscerosensory signals (Jarrahi et al., 2015) and to map cerebral circuits associated with clinical and behavioral features of functional gastrointestinal disorders (Qi et al., 2016; Sidhu et al., 2004), including IBS (Weng et al., 2017). In recent years, resting-state functional connectivity (FC) has emerged as a robust and sensitive metric for investigating neural network organization across various clinical populations (Park and Friston, 2013; van den Heuvel and Hulshoff Pol, 2010). Alterations in FC have been consistently observed in chronic pain conditions such as fibromyalgia (Kong et al., 2021), chronic back pain (Zhang et al., 2024), and migraines (Messina et al., 2022). Furthermore, accumulating rsFC studies in depression have consistently reported aberrant connectivity within the default mode network (DMN; Steffens et al., 2019), prefrontal cortex (PFC; Bekhbat et al., 2022), and sensorimotor network, core neural circuits involved in emotional regulation, self-referential processing, and sensory integration (Zhang Z. et al., 2025). For instance Wang et al. (2013), individuals with depressive symptoms exhibit reduced interhemispheric PFC connectivity and hyperconnectivity between the PFC and primary sensory cortex (SI), which are linked to emotional dysregulation and amplified interoceptive sensitivity. These rsFC abnormalities overlap with neural alterations observed in IBS, supporting a potential shared neural substrate between depressive symptoms and IBS pathophysiology (Park et al., 2018; Weng et al., 2017). Internalizing psychopathologies encompassing depression and anxiety, core affective comorbidities of IBS, are underpinned by a core set of shared cortical structural and functional alterations, most prominently in the prefrontal cortex (PFC), including reduced interhemispheric connectivity, impaired functional connectivity strength, and disrupted integration with limbic and sensorimotor networks (Kuang et al., 2023; Yu et al., 2023). Notably, distinct internalizing symptom subtypes also exhibit specific PFC subregional dysfunction: for example, depressive symptoms are closely linked to left-lateralized PFC functional deficits related to emotional regulation, whereas anxiety is associated with hyperconnectivity in the medial PFC involved in threat appraisal. These shared and specific cortical alterations form a critical neurobiological framework for understanding the high comorbidity of internalizing symptoms and IBS, yet how these PFC-centric changes translate to the unique brain-gut axis dysfunction in IBS remains unelucidated.

This study examined functional connectivity (FC) abnormalities in IBS and their correlations with the severity of both depressive and gastrointestinal symptoms. To uncover aberrant FC in patients, multiple analytic approaches were employed, including voxel-wise functional connectivity strength (FCS), seed-based whole-brain connectivity, and seed-to-seed FC analyses. We hypothesized that individuals with IBS would exhibit distinct alterations in brain FC, and that these patterns would be associated with the severity of both depressive symptoms and IBS-related clinical manifestations. Furthermore, we further hypothesized that aberrant rsFC mediates the relationship between depressive symptoms and IBS severity, reflecting a potential neural pathway linking affective disturbances to gastrointestinal symptom burden.

## Materials and methods

### Subjects

The study comprised 36 patients with irritable bowel syndrome (IBS) and 38 healthy controls (HCs), all of whom were right-handed. Each participant provided written informed consent prior to enrollment, in compliance with protocols approved by the local Medical Research Ethics Committee and consistent with the principles of the Helsinki Declaration. The IBS patients were recruited from the Digestive Disease Clinic of our hospital and were clinically diagnosed by a gastroenterologist specializing in functional gastrointestinal disorders according to the Rome III criteria. Healthy controls were recruited from the local community.

The diagnosis of IBS was established based on the presence of recurrent abdominal pain or discomfort accompanied by at least two of the following features: symptom relief following defecation, onset associated with altered stool frequency, or onset associated with changes in stool form. Exclusion criteria comprised a history of gastrointestinal surgery, psychiatric disorders, substance abuse, use of centrally acting agents (including selective serotonin reuptake inhibitors), use of any gastrointestinal medications (e.g., antispasmodics, laxatives, and prokinetics), or regular intake of aspirin or non-steroidal anti-inflammatory drugs for more than 2 weeks prior to enrollment. Additionally, individuals with significant medical or neurological illnesses, or those exhibiting head motion exceeding 1.0 mm in translation or 1.0° in rotation during MRI were excluded. Based on these criteria, three IBS patients and two healthy controls were excluded due to excessive head motion, and one IBS participant was removed for falling asleep during the scan, as self-reported afterward. The final analytical sample consisted of 32 IBS patients and 37 healthy controls, with the two groups matched for age, sex, and educational level.

All participants were evaluated using the Self-Rating Anxiety Scale (SAS) and the Self-Rating Depression Scale (SDS) to assess anxiety and depressive symptoms, respectively, in both IBS patients and healthy controls. Additionally, IBS patients completed the IBS-Symptom Severity Score (IBS-SSS), the IBS-Quality of Life (IBS-QOL) questionnaire, and a visual analog scale (VAS) for pain intensity, ranging from 0 (“no pain sensation”) to 10 (“the most intense pain sensation imaginable”).

### MRI data acquisition

All participants were scanned on a 3 Tesla MRI scanner (Siemens TIM Trio, Erlangen, Germany). To reduce head motion, foam padding was applied, and subjects were instructed to keep their eyes closed, remain awake, and avoid engaging in any specific thoughts. High-resolution T1-weighted structural images were first acquired sagittally using a magnetization-prepared rapid gradient-echo (MPRAGE) sequence with the following parameters: repetition time (TR) = 2,000 ms, echo time (TE) = 2.80 ms, flip angle = 11°, field of view (FOV) = 256 × 256 mm^2^, matrix = 256 × 256, slice thickness = 1 mm, and 191 sagittal slices. Subsequently, resting-state functional MRI (rs-fMRI) data were collected using a single-shot gradient-echo echo-planar imaging (EPI) sequence with the following settings: TR/TE = 2,000/30 ms, FOV = 260 × 260 mm^2^, flip angle = 78°, matrix size = 64 × 64, voxel size = 2.75 × 2.75 × 3 mm3, 44 axial slices aligned to the anterior-posterior commissure plane, total acquisition time = 10 min 30 s, yielding 315 volumes.

### Data preprocessing

Preprocessing of the functional imaging data was carried out using the Data Processing Assistant for rs-fMRI (DPARSF) toolbox. The first 10 volumes of each functional scan were discarded to allow for magnetic field stabilization and subject acclimatization. Head motion artifacts were corrected through realignment. The structural T1-weighted image was then co-registered to the mean functional image and spatially normalized to the Montreal Neurological Institute (MNI) template. Functional images were subsequently resampled to an isotropic resolution of 3 mm3. Nuisance covariates, including linear drift, global mean signal, white matter and cerebrospinal fluid signals, as well as the Friston 24-parameter model of head motion, were regressed out from the data. A scrubbing procedure was applied to remove volumes affected by excessive motion. Finally, temporal bandpass filtering (0.01–0.08 Hz) was performed to reduce high-frequency noise and low-frequency drift. The preprocessed data were used in all subsequent analyses.

### Functional connectivity (FC) analyses

Functional Connectivity Strength (FCS), along with seed-to-whole-brain and seed-to-seed FC analyses, were conducted to identify FC alterations in IBS patients. The resting-state fMRI data were processed using DPARSF to calculate FCS. For every participant, a whole-brain functional connectivity matrix was constructed by computing Pearson's correlation coefficients between the time series of every pair of voxels. These correlation coefficients were then converted to z-scores using Fisher's *r*-to-*z* transformation to ensure normality. The FCS for a given voxel was defined as the sum of the strengths (*z*-values) of its functional connections with all other brain voxels. A correlation threshold of *r* > 0.25 was applied during this computation to minimize the impact of spurious noise. This threshold is a widely used and validated criterion in resting-state fMRI functional connectivity strength analysis, which can effectively retain valid inter-voxel functional connections while suppressing non-neuronal signals and spurious noise interference (Li et al., 2016).

Subsequently, seed-based whole-brain FC analysis was performed. The mean time series were extracted from clusters showing significant between-group differences in the FCS analysis. These time series were then correlated with the time series of every other voxel in the brain to generate a whole-brain correlation map for each seed, which was also transformed into a *z*-map using Fisher's method.

For the seed-to-seed FC analysis, the mean time series were extracted from each of the 116 regions defined by the AAL atlas. A pairwise Pearson correlation analysis was then conducted between the time series of every pair of these regions to construct a subject-level connectivity matrix. The resulting correlation values were subsequently Fisher *z*-transformed for normalization. This seed-to-seed analysis involves all 116 brain regions defined by the AAL atlas, and the subsequent FDR correction for multiple comparisons was applied across all possible pairwise functional connections among these regions (i.e., 1161 × 15/2 = 6,670 edges in total).

### Statistical analyses

For each group, whole-brain FCS maps were analyzed using a random-effects one-sample *t*-test implemented in SPM12. Cluster-level family-wise error (FWE) correction was employed to control for multiple comparisons, with a cluster-defining voxel-level threshold of *p* = 0.001 and a corrected cluster significance level of *p* < 0.05. Between-group differences in FCS were examined *via* voxel-wise two-sample *t*-tests, including age, sex, and educational level as covariates of no interest. The same FWE correction procedure was applied at the cluster level. For seed-based whole-brain functional connectivity (FC), group differences were evaluated using voxel-wise two-sample *t*-tests, again controlling for age, sex, and education, with identical FWE correction criteria. In seed-to-seed analyses, pairwise FC values were computed per subject, and one-sample *t*-tests were conducted within each group. Multiple comparisons were addressed using false discovery rate (FDR) correction. Family-wise error (FWE) correction was adopted for FCS and seed-based whole-brain FC analyses because these voxel-level whole-brain analyses carry a high risk of false positive results, and FWE correction enables strict control of the overall Type I error rate (Flandin and Friston, 2019; Vandekar et al., 2018). Between-group comparisons of seed-to-seed FC were carried out using two-sample *t*-tests, including the same covariates, with FDR correction applied (*q* < 0.05). FDR correction was selected for seed-to-seed FC analysis due to the large number of pairwise connections (6,670 edges) in the analysis, which can balance false positive control and statistical power retention, a conventional strategy for large-scale connectivity matrix analysis (Dai et al., 2024; Li et al., 2008; Liu et al., 2012). To examine associations between altered FC and clinical symptoms in IBS, the mean rsFC value within a 6 mm spherical ROI centered on the cluster peak of significant ROIs obtained from between-group comparisons were extracted for Pearson correlation analyses with clinical scores. Based on the results of group and correlation analyses, mediation models were constructed to explore whether FC alterations mediated relationships between clinical variables. To empirically test our *a priori* hypothesis, we examined the hypothesized directional mediation pathway in which depression severity serves as the predictor, functional connectivity (FC) as the mediator, and IBS severity as the outcome. To further validate the specificity of the proposed mediation pathway, we examined the reverse model and additional exploratory models (e.g., direct and indirect effect decompositions) to rule out bidirectional mediation or spurious associations. This approach aligns with best practices for mediation analysis, ensuring that the observed effect is not confounded by reverse causality or unspecified pathways. Mediation effects were assessed using bootstrapping with 5,000 resamples *via* the PROCESS macro in SPSS, examining bias-corrected 95% confidence intervals. Effects were considered significant if the confidence intervals did not include zero.

## Results

### Demographic and clinical data

Demographic and clinical profile details were shown in Table 1. Specifically, the current study included 32 healthy controls and 37 IBS patients. No significant between group difference was observed between IBS and controls regarding age, years of education, and sex (*p* > 0.05). Clinical assessments, including the Self-Rating Anxiety Scale (SAS), Self-Rating Depression Scale (SDS), IBS-Symptom Severity Score, IBS-Quality of Life (IBS-QOL) score, and visual analog scale, revealed that patients with IBS exhibited a significant symptom burden and pain, along with higher levels of anxiety (*p* < 0.001) and depression (*p* < 0.001) compared to controls.

**Table 1:** Demographic and clinical variables for IBS patients and healthy controls.

Variables	IBS group	HC group	*P* value
Age	32.139 ± 0.17	32.211 ± 1.23	0.99
Sex (Male/Female)	18/19	16/16	0.98
Years of education	9.133 ± 0.24	9.112 ± 0.71	0.97
Duration of symptom	42.011 ± 1.32	NA	
SAS	42.139 ± 0.81	33.314 ± 0.14	**< 0.0001**
SDS	39.031 ± 2.66	33.174 ± 0.16	**0.0098**
IBS-SSS	161.166 ± 1.79	NA	
IBS-QOL	68.772 ± 2.15	NA	
VAS	4.143 ± 0.27	NA	

SAS, self-rating anxiety scale; SDS, self-rating depression scale; IBS-QOL, IBS-symptom severity score, IBS-quality of life score; VAS, visual analog scale.

### Functional connectivity strength (FCS)

**Figure 1:**
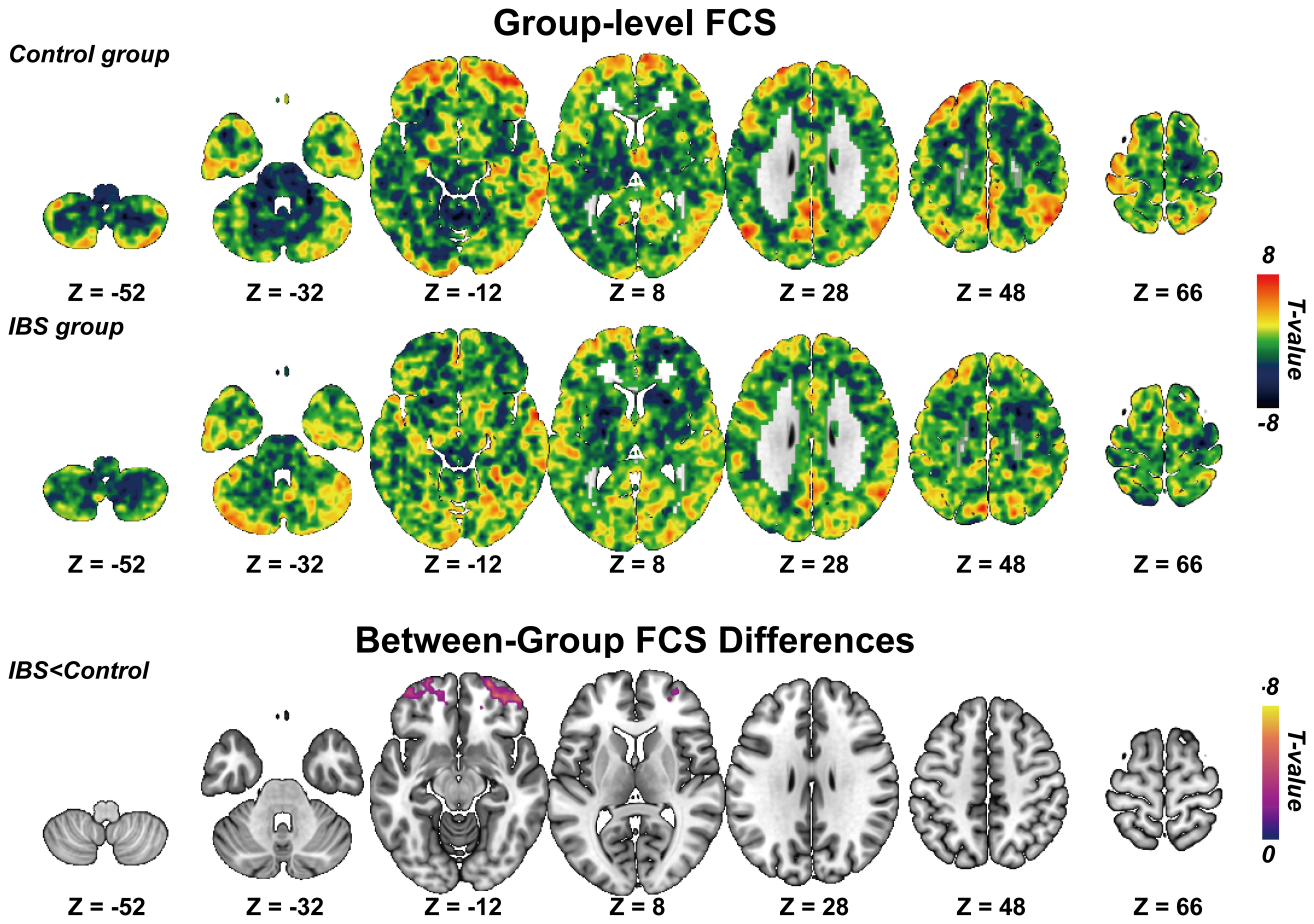
**(Upper, Middle)**. Group-level functional connectivity strength (FCS) revealed by one-sample *t* test and family-wise error (FWE) correction for control group and IBS group respectively. **(Lower)**. Between-group FCS differences between IBS and control group revealed by two-sample *t* test and FWE correction. IBS, irritable bowel syndrome.

Within-group analyses demonstrated a convergent pattern of functional connectivity strength (FCS) in both IBS patients and healthy controls. Specifically, both groups showed higher FCS within key nodes of the default mode network (DMN), including the medial prefrontal cortex, precuneus, and bilateral angular gyri (Figure 1, upper and middle panels). In between-group comparisons, the IBS group exhibited significantly reduced FCS relative to controls, particularly within the bilateral prefrontal cortices (Figure 1, lower panel; Table 2). These differences suggest alterations in prefrontal regulatory circuits in IBS.

**Table 2:** Brain regions exhibited significant between-group differences in functional connectivity strength between IBS patients and healthy controls (IBS-HC).

Brain regions	Maximal *T* score	Cluster size (voxels)	Peak location (*x,y,z*)
Left prefrontal cortex	−7.62	134	−24, 63, −15
Right prefrontal cortex	−8.17	314	39, 54, −15

### Seed-to-whole-brain functional connectivity

**Figure 2:**
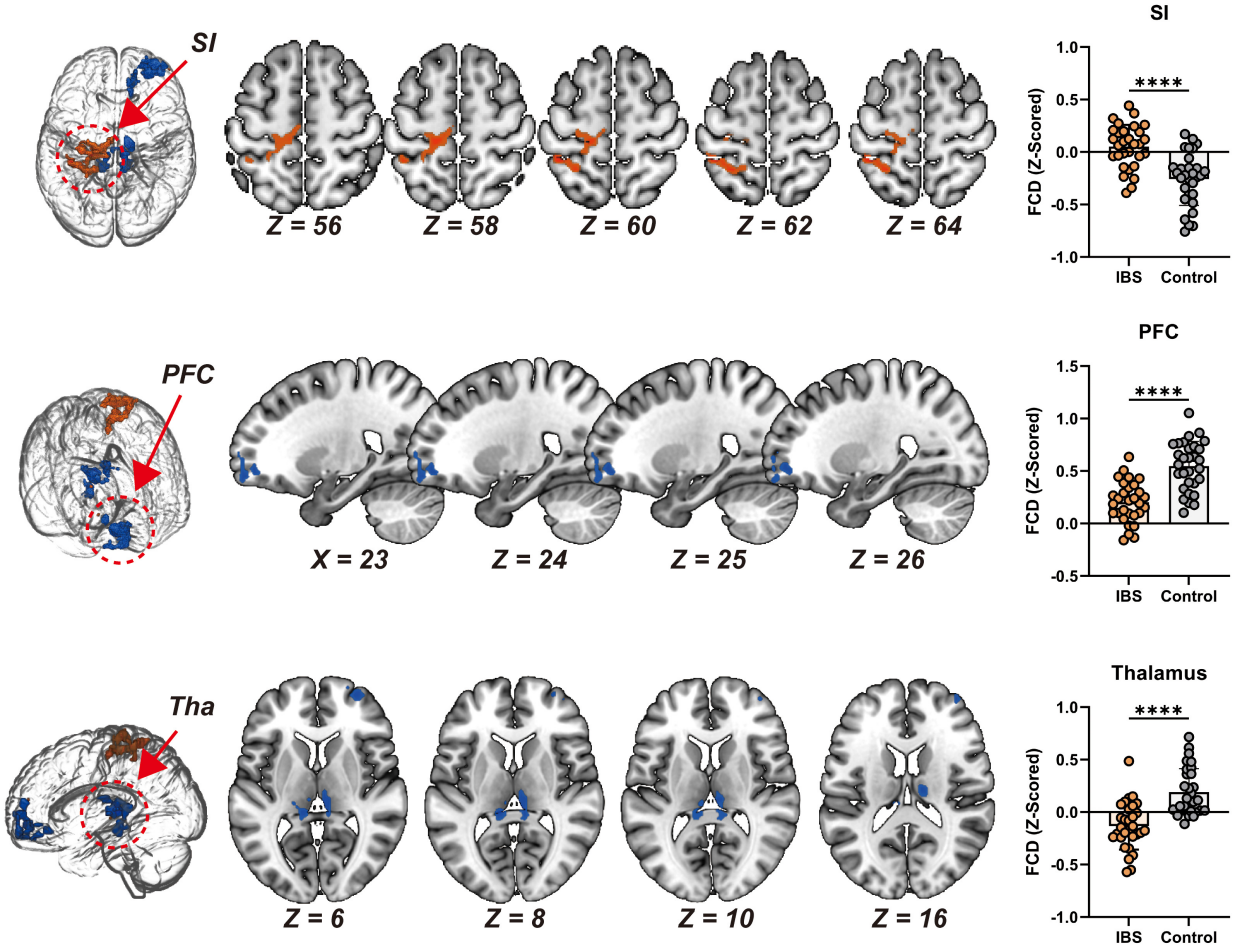
**(Left)** Spatial distribution of brain clusters exhibited significant differences in left prefrontal cortex to whole brain functional connectivity between IBS and control group. **(Right)** Bar plots for differences in left prefrontal cortex to whole brain functional connectivity between IBS and control group. SI, primary sensory cortex; PFC, prefrontal cortex; Tha, thalamus; IBS, irritable bowel syndrome. ^****^P < 0.001.

Following the FCS analysis, brain clusters demonstrating significant between-group differences between IBS patients and controls were selected as seeds for subsequent seed-based whole-brain functional connectivity (FC) analyses. Voxel-wise comparisons revealed that, compared to controls, the IBS group exhibited significantly higher FC between the left prefrontal cortex (PFC) and the primary sensory cortex (SI), but significantly lower FC between the left PFC and the right PFC, as well as between the left PFC and bilateral thalamus (Figure 2, Table 3). Analysis using the right PFC as a seed did not identify any significant group differences (FWE-corrected *p* > 0.05), indicating that rsFC alterations in IBS are more prominent in the left PFC-related circuits.

**Table 3:** Brain regions exhibited significant between-group differences in left prefrontal cortex to whole brain functional connectivity between IBS patients and healthy controls (IBS-HC).

Brain regions	Maximal *T* score	Cluster size (voxels)	Peak location (*x,y,z*)
Primary sensory cortex	5.17	135	−18, −36, 69
Right prefrontal cortex	−7.14	80	27, 48, −06
Bilateral thalamus	−5.17	61	9, −24, 6

Correlation analyses further indicated that the reduced FCS in the right PFC was significantly associated with weakened interhemispheric FC between the left and right PFC, suggesting that the observed reduction in right PFC FCS may primarily arise from impaired prefrontal homotopic connectivity. Similarly, the altered FCS in the left PFC was significantly correlated with enhanced FC between the left PFC and left SI, implying that the left PFC FCS changes are mainly driven by hyperconnectivity with the sensory cortex.

### Seed-to-seed functional connectivity

**Figure 3:**
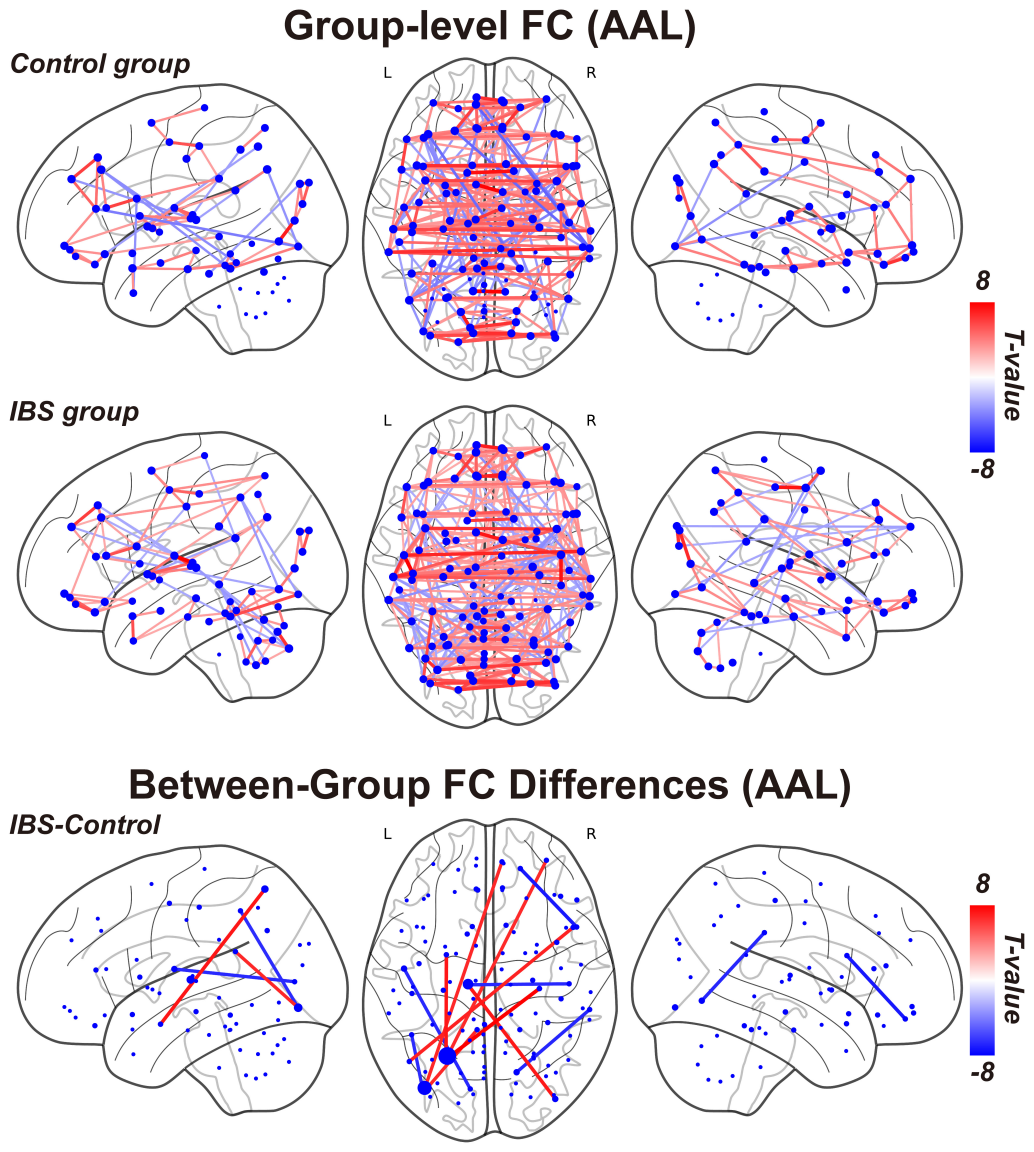
**(Upper, Middle)** Group-level seed-to-seed functional connectivity revealed by one-sample *t* test and false discovery rate (FDR) correction for control group and IBS group respectively. **(Lower)** Between-group seed-to-seed functional connectivity differences between IBS and control group revealed by two-sample *t* test and FDR correction. Node size represents the degree centrality of the brain region (a larger node size indicates a higher number of aberrant functional connections with other brain regions); the thickness of the connecting lines represents the magnitude of the *t*-value from the two-sample *t*-test for between-group differences in FC (a thicker line indicates a more significant group difference in functional connection). IBS, irritable bowel syndrome.

Using seed-based functional connectivity analysis with the AAL atlas, we observed strong interhemispheric connections within the sensorimotor network and the default mode network in both groups. Compared to controls, the IBS group exhibited 10 significantly higher and nine lower functional connections. Notably, the left angular gyrus emerged as a critical hub, demonstrating aberrant hyperconnectivity with multiple regions in IBS patients, including the right prefrontal cortex, left precentral gyrus, primary sensory cortex (SI), and left middle temporal gyrus (Figure 3).

### Correlation analyses

To further examine the link between abnormal functional connectivity (FC) and clinical characteristics, Pearson correlation analyses were performed. The mean rsFC value within a 6 mm spherical ROI centered on the cluster peak of significant ROIs were extracted for Pearson correlation analyses with clinical scores. A heatmap summarizes the correlation coefficients between altered FC metrics and clinical variables, including SAS, SDS, IBS-SSS, IBS-QOL, and pain VAS scores (Supplementary Figure S1). Supplementary Figure S1 shows that bilateral PFC FCS was not significantly correlated with SAS, IBS-QOL, or VAS scores (all *q* > 0.05). Specifically, FCS in the left PFC correlated negatively with SDS scores (*r* = −0.40, *q* = 0.049) and negatively with IBS-SSS (*r* = −0.48, *q* = 0.01). No significant correlations were observed between bilateral PFC FCS and SAS, IBS-QOL, or VAS ratings. Additionally, FC between the left PFC and SI was positively associated with pain intensity (VAS) in IBS patients (*r* = 0.44, *q* = 0.02). In contrast, FC between the left and right PFC was negatively correlated with SDS scores (*r* = −0.41, *q* = 0.049). No other seed-based FC values showed significant associations with clinical measures.

**Figure 4:**
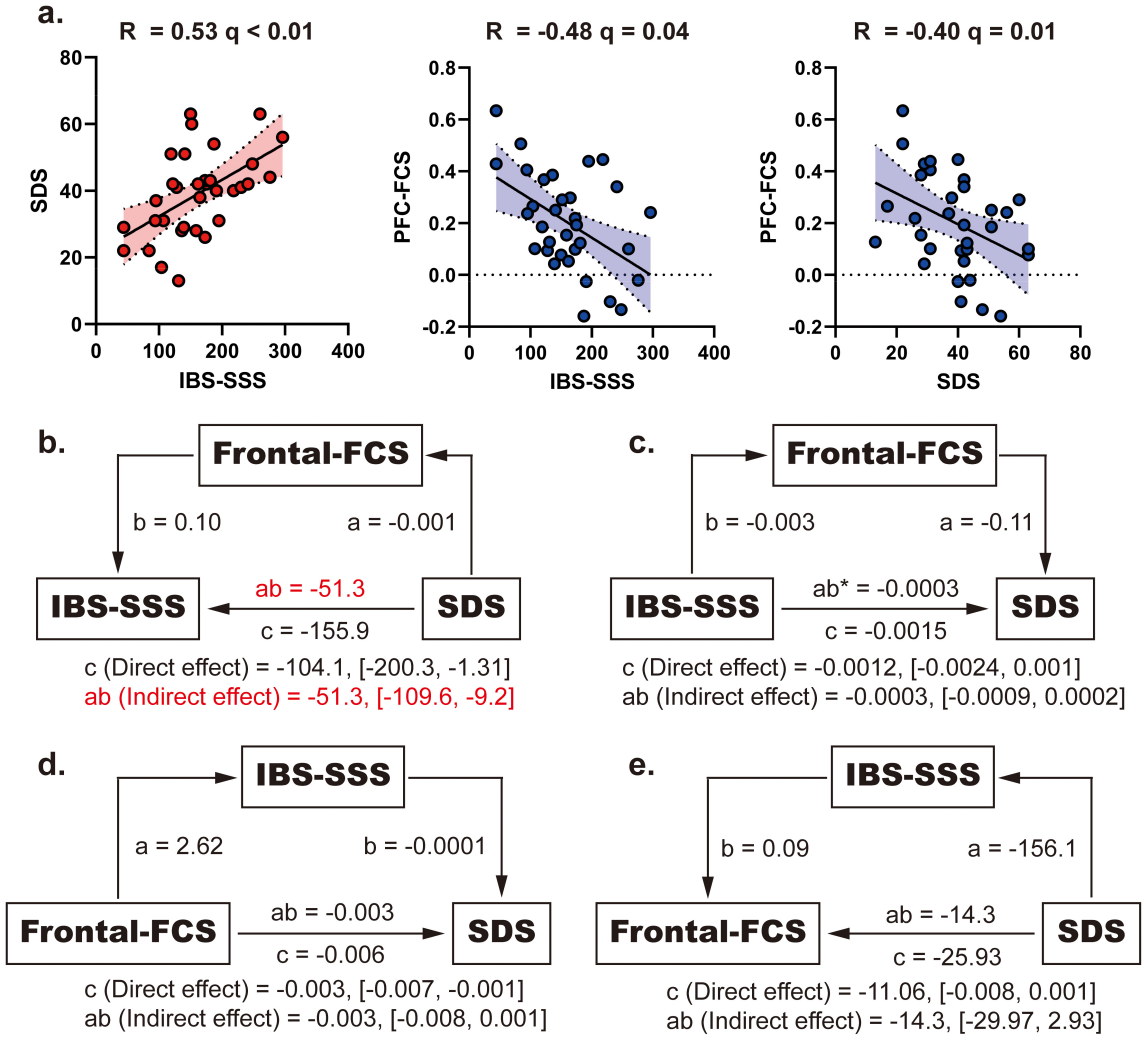
**(a)** Scatter plots for correlation among self-rating depression scale (SDS), left prefrontal cortex (PFC) functional connectivity strength (FCS) and IBS-symptom severity score (IBS-SSS) in irritable bowel syndrome (IBS) patients. **(b-e)** Mediation models for causal association between PFC FCS, IBS-SSS and SDS in IBS patients.

Of note, SDS and IBS-SSS scores were positively correlated with each other (*r* = 0.53, *q* < 0.01), indicating a clinical interplay between depressive symptoms and IBS severity. To assess causal pathways underlying these relationships, we conducted mediation analyses using bootstrapping with 5,000 iterations. A model specifying depression severity (SDS) as the independent variable, left PFC FCS as the mediator, and IBS-SSS as the outcome revealed a significant partial mediation effect [ab = −51.3, 95% CI (−109.6, −9.2); Figure 4], supporting a pathway in which depressive symptoms contribute to IBS severity *via* altered PFC FCS. Conversely, a reverse model with IBS-SSS as the predictor and SDS as the outcome did not show a significant mediation effect *via* PFC FCS [ab = −0.0003, 95% CI (−0.0009, 0.0002)], suggesting that this particular neural mechanism does not underlie the progression from gastrointestinal symptoms to depression, which may instead involve other circuits. It should be emphasized that this mediation finding is based on cross-sectional rs-fMRI data and is essentially correlational. Longitudinal follow-up designs are essential to establish the causal mechanism between these variables.

## Discussion

In the present study, we identified significant alterations in functional connectivity strength (FCS) in the prefrontal cortex (PFC), along with disrupted functional connectivity between the angular gyrus and multiple other brain regions. Both regions constitute integral hubs of the default mode network (DMN), a large-scale intrinsic network deeply involved in self-referential cognition, such as the continuous monitoring of internal bodily states and emotional awareness (Sanz-Morales and Melero, 2024; Smallwood et al., 2021; Zhang D. et al., 2025).

Internalizing psychopathologies such as depression and anxiety share core cortical alterations in the PFC, including reduced functional connectivity strength and impaired interhemispheric communication, while specific disease subtypes exhibit distinct regional PFC dysfunction characteristics (Kuang et al., 2023; Yu et al., 2023). These shared and specific cortical alterations form the neural basis for the high comorbidity of internalizing symptoms and IBS, and our findings of PFC functional connectivity alterations in IBS patients are highly consistent with this broader research framework of internalizing psychopathology. Accumulating evidence suggests that the DMN is among the neural networks most consistently altered in chronic pain conditions (Alshelh et al., 2018; Ng et al., 2021). Aberrant DMN connectivity has been frequently reported across a range of persistent pain disorders, including IBS (Icenhour et al., 2017; Kano et al., 2020). Our findings align with earlier work documenting abnormal DMN connectivity in IBS patients relative to healthy controls (Icenhour et al., 2017). Specifically, we further delineate these alterations by implicating the PFC and angular gyrus, two pivotal nodes that support the integration of interoceptive information with cognitive and affective functions (Charbonneau et al., 2024; Xu et al., 2014). The angular gyrus, in particular, acts as a hub integrating perceptual, attentional, and mnemonic information (Rockland, 2023; Seghier, 2013, 2023). Its decoupling from other regions, as observed herein, may signify deficient coordination between self-referential processing and sensory signals in IBS. Similarly, the PFC, especially its medial sectors, is centrally involved in autobiographical memory, emotional appraisal, and value-based decision-making (Block, 2024; Dixon et al., 2017; Kozuch, 2024). The observed FCS alterations within this region may reflect impaired communication among DMN subsystems, potentially underlying exaggerated self-focused attention and dysfunctional interpretation of visceral inputs.

Previous neuroimaging research in IBS has repeatedly identified abnormal activity and connectivity within these brain regions, both during aversive visceral stimulation and at rest. These observations further implicate DMN dysfunction in the pathophysiology of IBS, specifically pertaining to dysregulated neural processing of visceral information. Moreover, emerging evidence indicates that microstructural differences within DMN nodes, particularly those reflecting cytoarchitectonic variation, may contribute to variability in symptom presentation and cognitive-affective burden across patients. The DMN is not a monolithic structure; rather, it consists of subregions with differing degrees of sensory integration autonomy. For example, the anterior medial prefrontal cortex is mainly involved in internalizing attention and emotional appraisal, while the posterior cingulate cortex is core to self-referential thinking and interoceptive processing; the functional heterogeneity of these subregions leads to distinct psychophysiological processes when their connectivity is disrupted in IBS patients with depressive symptoms. In addition, the sensorimotor network also exhibits obvious functional heterogeneity, and the abnormal FC between the PFC and the primary sensory cortex (SI) observed in our study is specifically associated with visceral hypersensitivity and central pain processing, which is different from the functional changes of other subregions in the sensorimotor network related to motor control. Certain areas are more receptive to external stimuli, while others, such as those in the medial prefrontal and posterior cingulate cortex, form a comparatively insulated core. This organizational principle may account for the diverse symptomatology associated with DMN disturbances, including visceral hypervigilance and persistent ruminative thoughts (Ji and Xia, 2022). Our results support the growing consensus that IBS pathophysiology extends beyond peripheral mechanisms to include distributed network disruptions that integrate visceral perception with cognitive and emotional processing.

Our findings also showed decreased inter-hemisphere FC in IBS patients compared with the control group. Recent studies on hemispheric asymmetry and interhemispheric interactions in psychiatric outcomes have demonstrated that impaired interhemispheric PFC connectivity leads to abnormal functional asymmetry of the prefrontal lobe (Fan et al., 2023; Wu et al., 2025), which further causes defective emotional regulation by disrupting the balanced processing of emotional information between the two hemispheres. The reduced interhemispheric FC between the left and right PFC observed in our study may reflect the imbalance of hemispheric emotional processing in IBS patients with depressive symptoms, which is a key neural basis for their emotional dysregulation. Alterations were also observed within the sensorimotor network, where IBS patients exhibited strengthened functional connectivity (FC) between the prefrontal cortex (PFC) and primary somatosensory cortex (SI) compared to controls. As the interoceptive cortex, SI is pivotal in representing the body's physiological condition and integrating multimodal sensorimotor information, particularly related to visceral sensation and pain processing (Hamdy et al., 2002). This aligns with recent observations of heightened neural responses to visceral distention in hypersensitive patients (Grinsvall et al., 2021). The elevated FC within this network may therefore reflect amplified ascending visceral inputs and heightened central processing of interoceptive signals in IBS. This interpretation is supported by a positive correlation between PFC–SI connectivity and subjective pain ratings among patients. These results extend earlier reports linking visceral hypersensitivity to clinical severity by identifying resting-state FC of the sensorimotor network as a potential neural substrate of this relationship (Weng et al., 2017). Additionally, patients showed reduced FC between the thalamus and PFC. As a central hub of the homeostatic afferent pathway, the thalamus relays sensory information from the brainstem to cortical regions such as the insula and mid-cingulate cortex, thereby modulating affective, motivational, and motor responses to stimuli. Convergent evidence indicates thalamic dysfunction in IBS across various paradigms, including visceral distension and thermal stimulation. For instance, Ke et al. (2015) reported abnormal regional homogeneity (ReHo) within the thalamus in IBS patients using resting-state fMRI, suggesting impaired local synchronization (Ke et al., 2015). Similarly, Qi et al. (2016) identified altered interhemispheric coordination in the thalamus using voxel-mirrored homotopic connectivity (Qi et al., 2016). Thus, the current observation of disrupted thalamocortical connectivity between thalamus and PFC not only corroborates earlier findings but also provides further insight into the thalamic contribution to aberrant visceral processing in IBS.

Building upon the evidence of DMN impairment in IBS, our findings further establish a robust and clinically significant link between depressive symptoms and IBS severity. Specifically, the degree of altered prefrontal FCS was correlated with both increased depressive affect and greater gastrointestinal symptom burden. Notably, mediation analysis revealed that prefrontal FCS mediates the relationship between depression and IBS severity, suggesting that dysregulation within this DMN node serves as a central mechanism through which depressive states exacerbate visceral hypersensitivity. These results carry two primary clinical implications. First, they emphasize the importance of recognizing and addressing depressive symptoms in IBS treatment, not merely as a comorbid condition, but as a contributor to underlying neural and visceral dysregulation. Second, the identified mediating role of prefrontal FCS highlights the left angular gyrus and bilateral PFC (key aberrant hubs of the DMN) as a promising target for neuromodulatory interventions. Emerging studies suggest that non-invasive brain stimulation techniques, such as personalized transcranial magnetic stimulation, may help normalize aberrant connectivity patterns and alleviate both depressive and pain symptoms in various clinical populations. Practical challenges in using these regions as stimulation targets include the accurate localization of individual brain functional hubs (due to interindividual variability in brain structure and function) and the optimization of stimulation parameters (e.g., frequency, intensity, and duration) for IBS patients with different depressive symptom severities. Corresponding optimization strategies can include combining individual rs-fMRI data for personalized target localization and conducting dose-response studies to determine the most effective stimulation parameters. In addition, repetitive transcranial magnetic stimulation (rTMS) targeting the left PFC has shown potential in improving emotional regulation and visceral hypersensitivity in preliminary studies, which provides a feasible translational direction for clinical intervention of IBS with depressive comorbidities. Future longitudinal research should examine whether antidepressant-induced normalization of prefrontal FCS occurs before, concurrent with, or after reductions in IBS severity, thereby elucidating the temporal dynamics of these network-level adaptations.

## Limitations

Current study has several limitations: a notable limitation is the exclusion of participants with diagnosed psychiatric disorders, resulting in the assessment of sub-clinical depressive symptoms only. SDS scores in both groups were within the normal range, which may limit the generalizability of our findings to IBS patients with clinical depression. Future studies should include individuals with comorbid clinical depression to validate the neural pathway identified herein. The current study focused on functional connectivity; structural connectivity measures are also needed; Future research should also consider the potential impact of other confounding variables, such as lifestyle factors, comorbidities, genetic predispositions, and the menstrual cycle phase of female participants that may influence cognitive health and visceral pain perception in IBS patients, which may be a potential confounding variable. Furthermore, expanding the sample size and diversity of participants would strengthen the generalizability of our findings, making the results more applicable to the broader population of IBS. The current analytical sample (32 IBS patients and 37 healthy controls) is relatively modest, which may bring potential risks of Type II errors or overfitting in complex bootstrapping mediation analyses and whole-brain voxel-wise connectivity analyses. We have adopted strict multiple comparison correction strategies (FWE and FDR) in all analyses to minimize the above risks, and future research with expanded sample size and multi-center cohorts is needed to conduct confirmatory analyses and improve the statistical power of the findings. Future research adopts a longitudinal design with repeated rs-fMRI scans is needed to verify the temporal dynamics of the relationship between depressive symptoms, left PFC FCS alterations and IBS severity, which is the key to further confirming the directional causal pathway of the brain-gut axis in IBS. In addition, this study only utilized resting-state fMRI, which can only reflect the intrinsic functional connectivity of the brain at rest; future research combining task-based fMRI (e.g., visceral stimulation, emotion regulation tasks) will more directly reveal the dynamic activities of the identified DMN and sensorimotor network hubs and their real-time associations with clinical symptoms, thereby enhancing the explanatory power of the study findings.

## Conclusion

Based on the present findings, patients with IBS demonstrate altered brain functional connectivity, characterized by reduced prefrontal functional connectivity strength. Furthermore, depressive symptoms partially mediate IBS severity through changes in prefrontal function, suggesting a potential pathway by which affective disturbances influence gastrointestinal symptomatology *via* top-down regulatory mechanisms.

## Data Availability

The raw data supporting the conclusions of this article will be made available by the authors, without undue reservation.
