# Supplementary material for: Disrupted prefrontal-sensorimotor functional connectivity mediates the association between depression and symptom severity in patients with irritable bowel syndrome
Source: Front Hum Neurosci. 2026 Apr 14;20:1817128. doi: 10.3389/fnhum.2026.1817128 (PMC13121372; doi:10.3389/fnhum.2026.1817128)
Supplement: Supplementary file 1 [file Data_Sheet_1.docx]

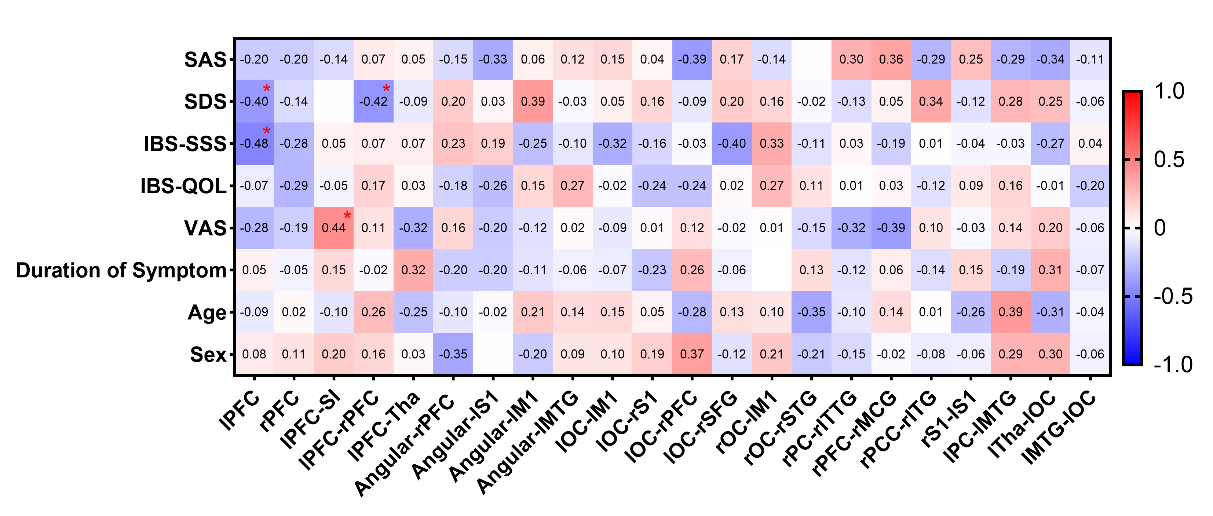


**Figure S1.** Correlation heatmap for association between clinical variables and functional connectivity changes in irritable bowel syndrome patients. *q<0.05, FDR-corrected
